# GPX4 overexpressed non-small cell lung cancer cells are sensitive to RSL3-induced ferroptosis

**DOI:** 10.1038/s41598-023-35978-9

**Published:** 2023-05-31

**Authors:** Joo-Won Kim, Dong Wha Min, Dasom Kim, Joohee Kim, Min Jung Kim, Hyangsoon Lim, Ji-Yun Lee

**Affiliations:** 1 Department of Pathology, Korea University College of Medicine, 73, Goryeodae-ro, Seongbuk-gu, Seoul, 02841 South Korea; 2 Department of Biomedical Science, Korea University College of Medicine, 73, Goryeodae-ro, Seongbuk-gu, Seoul, 02841 South Korea; 3 Department of Biological Sciences, Sookmyung Women’s University, Seoul, 04310 South Korea

**Keywords:** Cancer, Cell biology, Drug discovery, Molecular biology, Biomarkers, Molecular medicine, Oncology

## Abstract

Ferroptosis can be induced by inhibiting antioxidant enzymes GPX4 or system Xc^−^, increased intracellular iron concentrations, and lipid peroxidation. Recently, it has been suggested that ferroptosis can be an effective way to induce cancer cell death, although the specific relevance and mechanism of ferroptosis have not been fully elucidated. Here, we investigated the anticancer effects of ferroptosis inducers erastin and RSL3 on non-small cell lung cancer (NSCLC) cells. RSL3 induced cell death more effectively in NSCLC cells than erastin, with limited cytotoxicity in BEAS-2B normal bronchial epithelial cells. The sensitivity of NSCLC cells to RSL3 induced death was dependent on GPX4 expression levels; the effect of RSL3 was reversed by ferrostatin-1 (a ferroptosis inhibitor) but not by Z-VAD-FMK, chloroquine, bafilomycin A1, or necrostatin-1. RSL3 induced ferroptosis by promoting lipid peroxidation, elevating intracellular LIP concentration and ROS level, and blocking GSH-to-GSSH conversion through the inhibition of GPX4 and induction of Nrf2/HO1. Furthermore, RSL3 induced autophagosomes but disrupted the formation of autolysosomes with lysosomal membrane destabilization. GPX4 knockdown had a similar effect on ferroptosis phenotypes as RSL3. Taken together, RSL3-induced ferroptosis depends on the regulation of GPX4-Nrf2/HO1 in NSCLC cells. These results may be useful in predicting the ferroptosis response in NSCLC as well as drug resistant cancer cells.

## Introduction

Ferroptosis is a novel type of iron-dependent, oxidative damage-related cell death that is distinguished from other types of programmed cell death based on its genetic, morphological, and biochemical characteristics^1^. Ferroptosis is linked to the accumulation of lipid peroxides and iron, which can be caused by the failure of antioxidant activity through lipid peroxidation. The most established cause of lipid peroxidation is the regulation of cellular levels of glutathione (GSH) via the SLC7A11/xCT and the antioxidant enzyme glutathione peroxidase 4 (GPX4) axis. SLC7A11/xCT is a transporter subunit of system Xc^−^ that imports cystine in exchange for glutamate in an ATP-independent manner. Once imported into the cell by SLC7A11, cystine is reduced to cysteine via nicotinamide adenine dinucleotide phosphate (NADPH) consumption^2,3^. Cysteine is then used to produce GSH, which is oxidized to glutathione disulfide (GSSH) by GPX4 to reduce hydrogen peroxide, organic hydroperoxides and lipid hydroperoxides. Thus, the inhibition of cystine import by SLC7A11 limits GSH synthesis, which indirectly reduces GPX4 activity. Direct GPX4 inhibition reduces antioxidant capacity and results in the accumulation of lipid ROS, leading to oxidative damage and the promotion of ferroptosis^4–7^.

Ferroptosis was originally recognized in mutant RAS-harboring cancer cells by studying the effects of the small molecules such as erastin, RSL3, and other related compounds/agents^8–10^. Since then, research has been conducted using various ferroptosis inducers, including erastin as an SLC7A inhibitor and RSL3 as a GPX4 inhibitor, to explore the anticancer effects of these compounds regardless of RAS mutation and to overcome therapy resistance in various cancer cells^11–20^. Further investigations to understand the regulatory pathways and hallmarks associated with ferroptosis suggest that various molecules such as p53, Nrf2/Keap1, Nrf2/HO1, Yap/TAZ, and TFR1, along with SLC7A11 and GPX4, play important roles in the process and function of ferroptosis^4–6,11–14,21–26^. Thus, targeting ferroptosis as a novel strategy for cancer therapy requires elucidation of its mechanism of action in various cancers. However, few studies have investigated the anticancer effects of erastin and RSL3 in lung cancer^16,27–30^.

In this study, we investigated and compared the sensitivity to RSL3 and erastin in non-small cell lung cancer (NSCLC) and normal bronchial epithelial cells, BEAS-2B. RSL3 sensitivity varied in different NSCLC cell lines, and it caused limited cytotoxicity in BEAS-2B cells; however, erastin showed resistance to cell death in NSCLC cells along with cytotoxicity in BEAS-2B cells. The factors involved in the sensitivity to RSL3 were examined and further confirmed using an in vivo zebrafish model.

## Results

### Sensitivity to RSL3 was associated with GPX4 expression level in NSCLC cells

**Figure 1:**
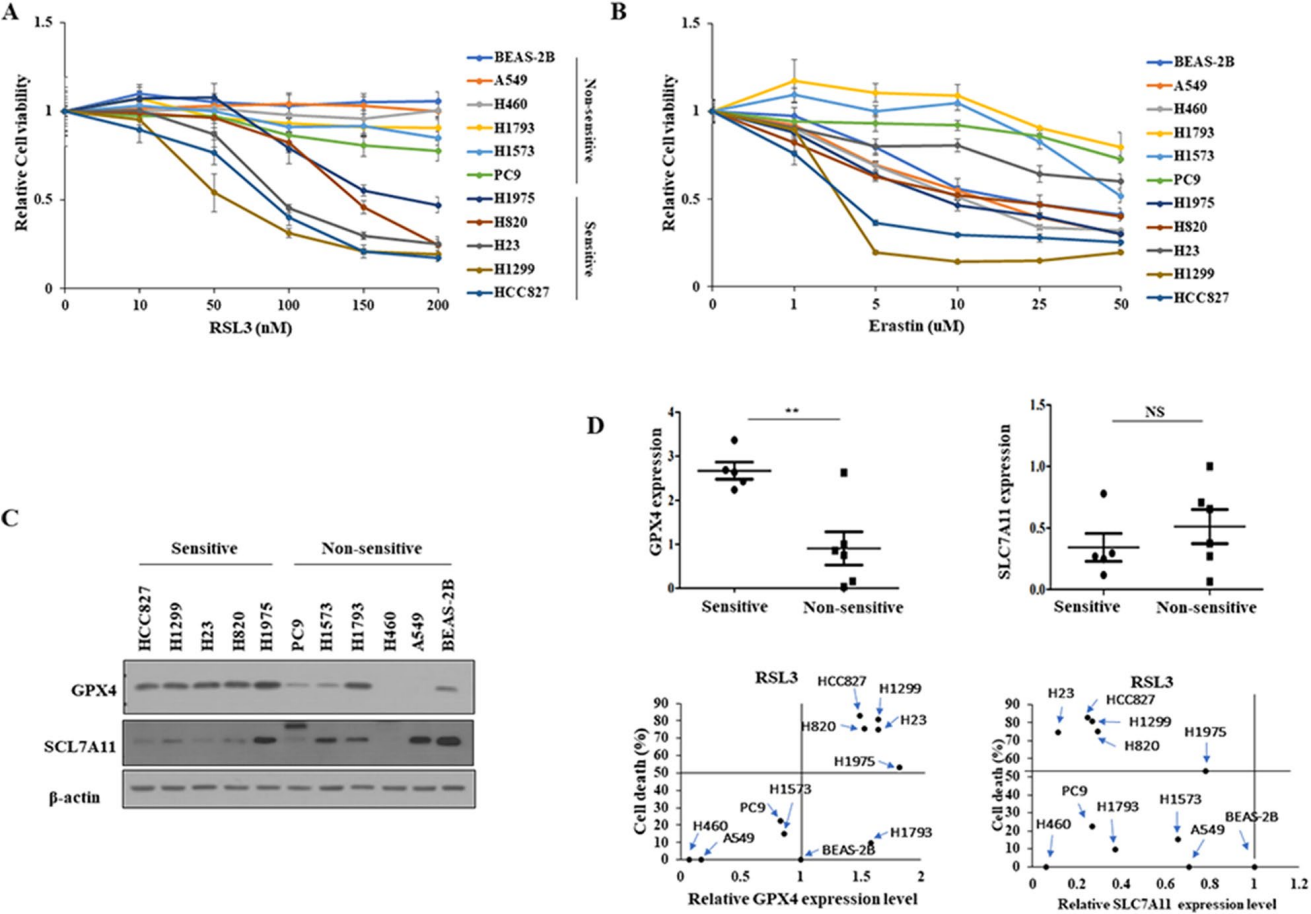
Sensitivity of NSCLC cells to RSL3 was associated with endogenous GPX4 expression level. Cell viability was determined using MTT assay following treatment with (**A**) RSL3 or (**B**) erastin for 48 h. Cells were divided into sensitive and non-sensitive groups, based on RSL3 responsiveness. (**C**) Expression levels of ferroptosis-associated proteins, GPX4 and SLC7A11, were examined via western blotting. (**D**) Correlations between the expression of GPX4 or SLC7A11, and the sensitivity of NSCLC cells to RSL3 (sensitive, *n* = 5; non-sensitive, *n* = 6). Results are shown as the mean ± SD of triplicate experiments. *p < 0.05, **p < 0.01, ***p < 0.001 compared with control.

To investigate whether ferroptosis inducers have anticancer effects on NSCLC cells, the effects of the GPX4 inhibitor RSL3 and the SLC7A11 inhibitor erastin on cell viability were evaluated in various NSCLC cells, as well as in normal bronchus cells, BEAS2-B, using the MTT assay (Table 1). NSCLC cells were divided into two groups based on cell viability in response to RSL3: H1975, H820, H23, H1299, and HCC827, which belong to the sensitive group; and A549, H460, H1793, H1573, and PC9, which belong to the non-sensitive group. Moreover, RSL3 had limited cytotoxic effects on BEAS-2B cells (Fig. 1A). However, the effect of erastin was divergent, and BEAS-2B cells were relatively sensitive to erastin when compared to NSCLC cells (Fig. 1B). Further investigations focused only on RSL3. Next, the effect of RSL3 on the expression of the ferroptosis markers, GPX4 and SLC7A11, was examined. Interestingly, only GPX4 expression, and not SLC7A11 expression, was significantly associated with RSL3 sensitivity in NSCLC cells (Fig. 1C,D).

**Table 1:** IC_50_ values of RSL3, and erastin after 48 h in BEAS2-B, and NSCLC cells.

	BEAS-2B	A549	H460	H1793	H1573	PC9	H1975	H820	H23	H1299	HCC827
RSL3 (nM)	> 200	> 200	> 200	> 200	> 200	> 200	182	144	95	59	87
Erastin (µM)	20	15	11	> 50	> 50	> 50	9	17	> 50	3	4

IC_50_, The half maximal inhibitory concentration.

### Ferroptosis was induced in RSL3 sensitive NSCLC cells by inhibition of GPX4, and induction of KEAP1/Nrf2-HO1

**Figure 2:**
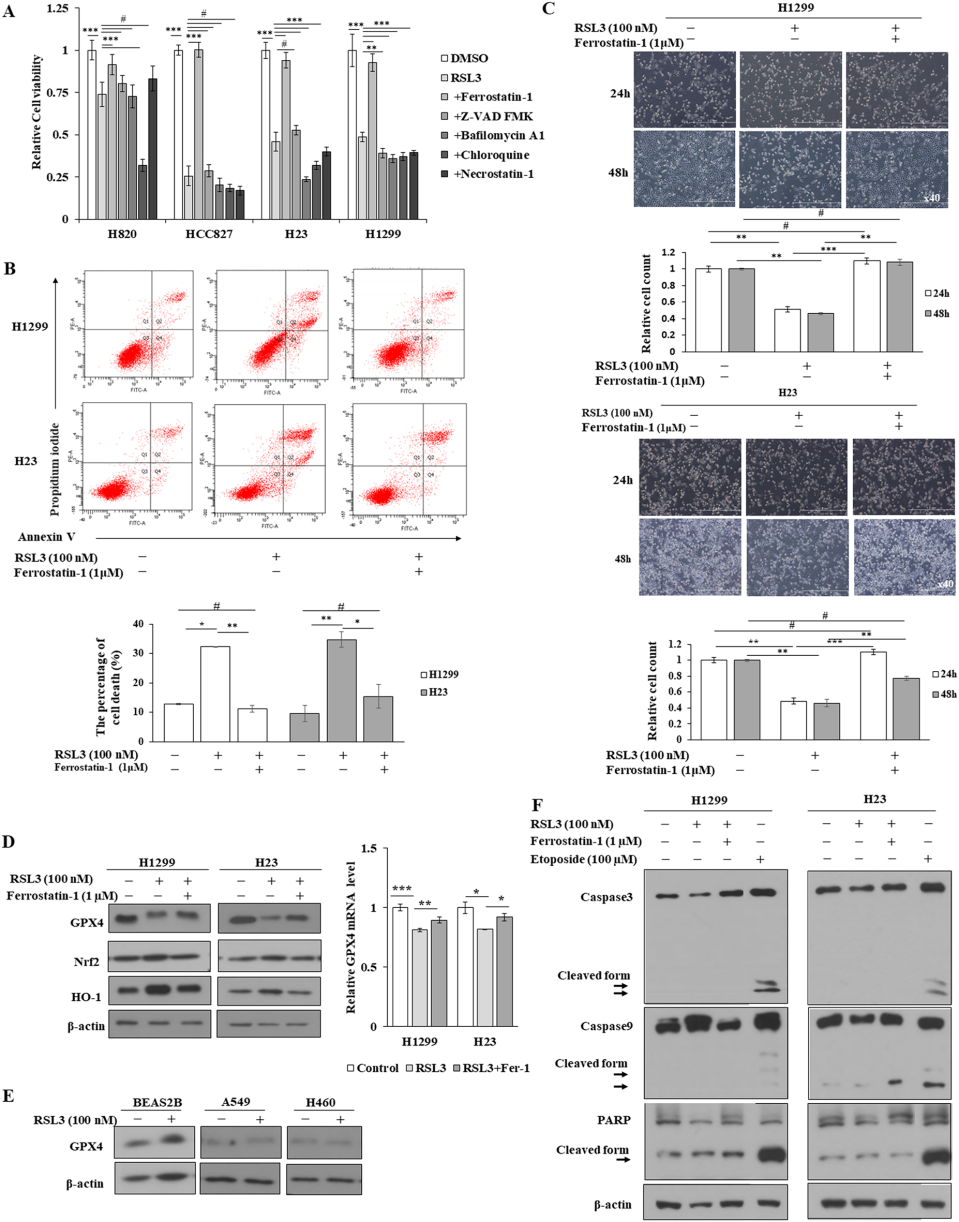
RSL3-induced cell death in RSL3-sensitive NSCLC cells was rescued by ferrostatin-1. Cells were treated with RSL3 with or without ferrostatin-1, Z-VAD FMK (5 µM), bafilomycin A1 (5 nM), chloroquine (50 µM), or necrostatin-1 (1 µM). (**A**) Cell viability measurement using the MTT assay. (**B**) FACS analysis with Annexin V/PI staining for examining cell death. (**C**) Cell proliferation measurement by cell counting. Representative cells images were captured by a light microscope (scale bar: 1 mm). (**D**–**F**) Expression of indicated molecules was detected by western blotting or qRT-PCR. Results are shown as the mean ± SD of triplicate experiments. *p < 0.05, **p < 0.01, ***p < 0.001 compared with control.

To verify whether RSL3-induced cell death was due to ferroptosis, the effect of RSL3 co-treatment with ferrostatin-1, a pharmacological ferroptosis inhibitor; Z-VAD-FMK, a pan-caspase inhibitor; chloroquine; bafilomycin A1, an autophagy inhibitor; and necrostatin-1, RIP kinase inhibitor, on cell death and proliferation of NSCLC cells was examined using MTT, flow cytometry, and cell counting. The results showed that RSL3-induced cell death was not rescued by Z-VAD-FMK, chloroquine, bafilomycin A1, or necrostatin-1 co-treatment (Fig. 2A), but it was rescued by ferrostatin-1 (Fig. 2A–C). Since GPX4 expression is associated with RSL3-induced cell death, the effect of RSL3 treatment on GPX4 expression was examined using qRT-PCR and western blotting. RSL3 suppressed GPX4 expression, which was restored by cotreatment with ferrostatin-1 (Fig. 2D). In addition, Nrf2/HO1 examination showing increase of Nrf2 and HO1 levels by RSL3, which were restored by ferrostatain-1 (Fig. 2D). However, GPX4 expression was not suppressed by RSL3 in RSL3-non-sensitive NSCLC cells (Fig. 2E). Furthermore, RSL3 treatment did not cause cleavage of apoptosis-related proteins such as caspase-3, cleaved caspase-9, and PARP; however, cleavage was evident with the apoptosis inducer etoposide (Fig. 2F). Taken together, these results show that the type of cell death induced by RSL3 treatment in NSCLC cells is ferroptosis, and that RSL3 also causes the suppression of GPX4 and induction of the Nrf2/HO1 pathway.

### RSL3 disrupted formation of autolysosome with lysosomal membrane destabilization

**Figure 3:**
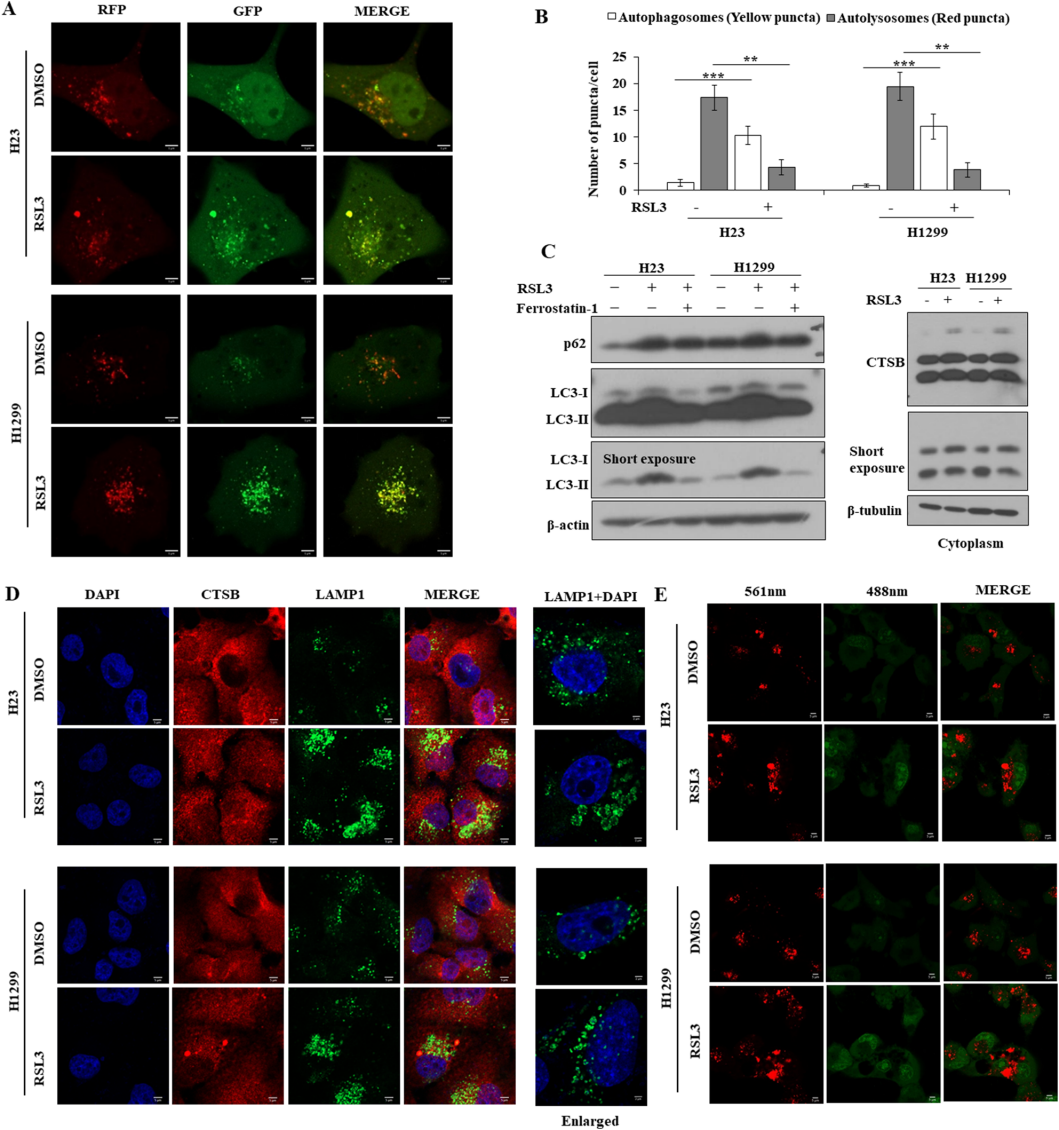
RSL3 disrupted formation of autolysosome by lysosome membrane destabilization. RSL3 (100 nM) treatment for 24 h. (**A**) Cells were treated with RSL3 after transfection of mRFP-GFP-LC3 plasmid. (**B**) The number of GFP-LC3B puncta/cell was calculated. (**C**) Expression of indicated molecules was detected by western blotting after treatment of RSL3, with or without ferrostatin-1 (1 µM). (**D**) Representative image was captured by confocal microscope after immunofluorescence staining with CSTB (red), LAMP1 (green), and DAPI (blue) (scale bar: 5 µm). Enlarged image (scale bar: 2 µm). (**E**) Representative fluorescence live cell image after staining with acridine orange was captured by laser confocal microscope (scale bar: 5 µm). Results are shown as the mean ± SD of triplicate experiments. *p < 0.05, **p < 0.01, ***p < 0.001 compared with control.

The association between autophagy and ferroptosis is known, and autophagic flux was examined using the mRFP-GFP-LC3 tandem plasmid, with yellow puncta indicating autophagosomes and red puncta indicating autolysosomes. The results showed that the number of autophagosomes increased, but that of autolysosomes decreased, indicating the disruption of autolysosome formation (Fig. 3A,B). Western blotting of the autophagy markers LC3 and p63 showed the accumulation of both LC3-II and p62 after treatment with RSL3, indicating that autophagy was induced; however, p62 was not degraded (Fig. 3C), indicating that autophagic flux was disrupted. To assess the effects of RSL3 on lysosomes, immunofluorescence staining with cathepsin B (CTSB), LAMP1, and acridine orange, and western blotting with cathepsin B were performed. Cathepsin B diffused throughout the cytoplasm and LAMP1 showed enlarged lysosomes (Fig. 3C,D). Acridine orange staining showed an increased massive red signal, indicating that the acidic compartment was localized in the cytoplasmic perinuclear organelles, suggesting a lysosomotropic effect of RSL3 (Fig. 3E). These results indicated destabilization of the lysosomal membrane by RSL3.

### RSL3 increased ferroptosis-associated LIP concentration, lipid peroxidation, ROS level, and GSH/GSSG ratio

**Figure 4:**
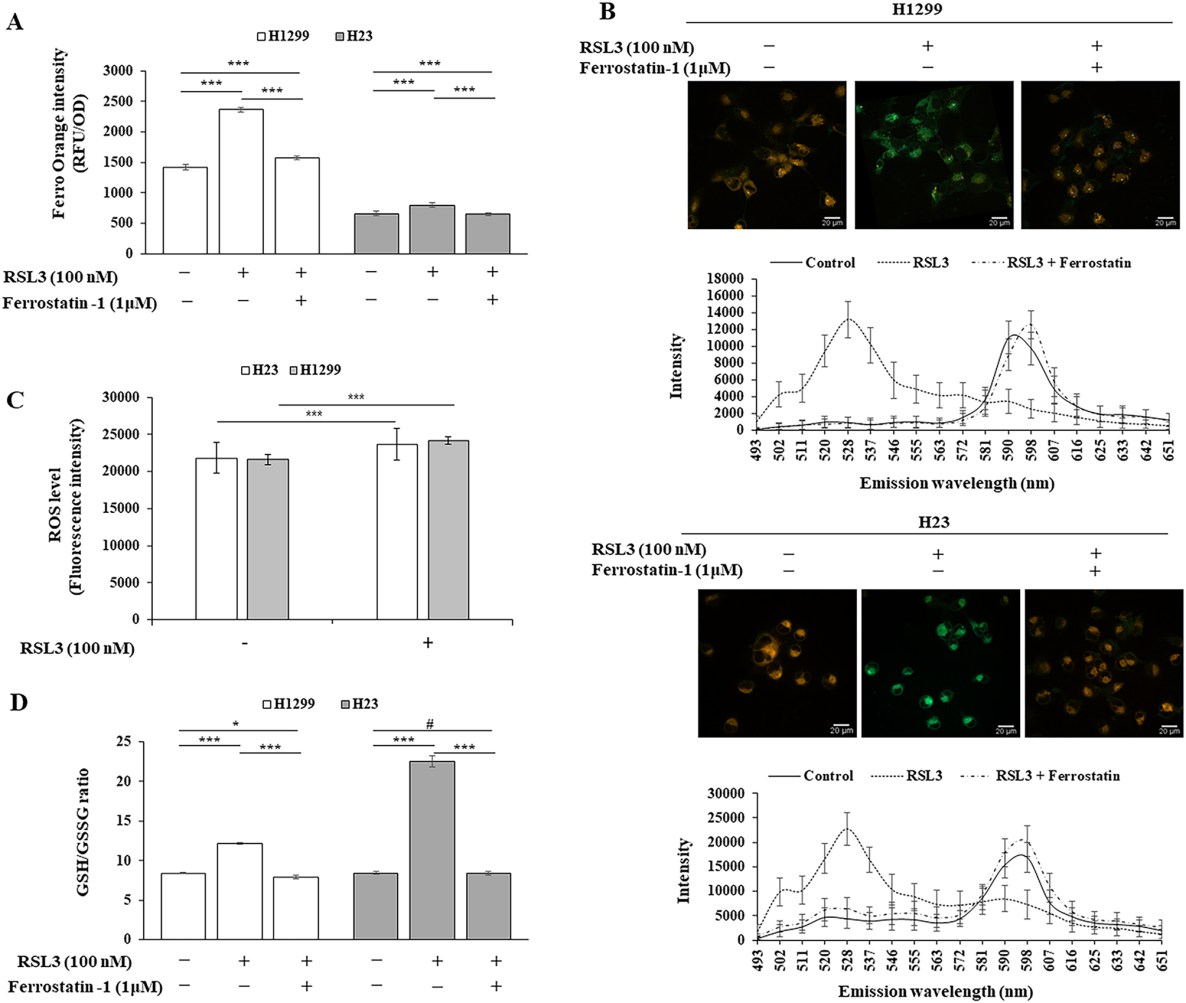
RSL3 promoted ferroptosis-associated characteristics. Cells were treated with RSL3, with or without ferrostatin-1 for 24 h (**A**, **B**, **D**). (**A**) Intracellular LIP expression level measured by the fluorescence intensity of FerroOrange. (**B**) Lipid peroxidation detected by BODIPY 581/591 C11 staining (scale bar: 20 µm). (**C**) Cellular ROS level determined by DCFA/H2DCFDA-cellular ROS assay kit after treatment with RSL3 for 6 h. (**D**) GSH/GSSH ratio determined by an EZ-Glutathione assay kit. Results are shown as the mean ± SD of at least triplicate experiments. *p < 0.05, **p < 0.01, ***p < 0.001 compared with control.

Increased intracellular LIP concentration, commonly observed during ferroptosis induction, causes lipid peroxidation by generating cellular reactive oxygen species (ROS). Therefore, the effects of RSL3 alone and RSL3 co-treatment with ferrostatin-1 on intracellular LIP levels and lipid peroxidation activity were examined by measuring the fluorescence intensity of FerroOrange and BODIPY 581/591 C11 staining in H1299 and H23 cells. As expected, RSL3 treatment increased LIP levels and co-treatment with ferrostatin-1 blocked this effect (Fig. 4A). In addition, RSL3 caused lipid peroxidation, as shown by C11-BODIPY stained cells that oxidized to form C11-BODIPY. This effect was reversed by ferrostatin-1 co-treatment (Fig. 4B). Increased cellular ROS levels after treatment with RSL3 were further confirmed by the DCFDA assay (Fig. 4C). Furthermore, the GSH/GSSH ratio was increased by RSL3 treatment and was reversed by ferrostatin-1 co-treatment (Fig. 4D). Together, these results confirmed that RSL3 increased LIP levels, lipid peroxidation, and the GSH/GSSG ratio, which are characteristics of ferroptosis.

### GPX4 knockdown showed effects similar to RSL3

**Figure 5:**
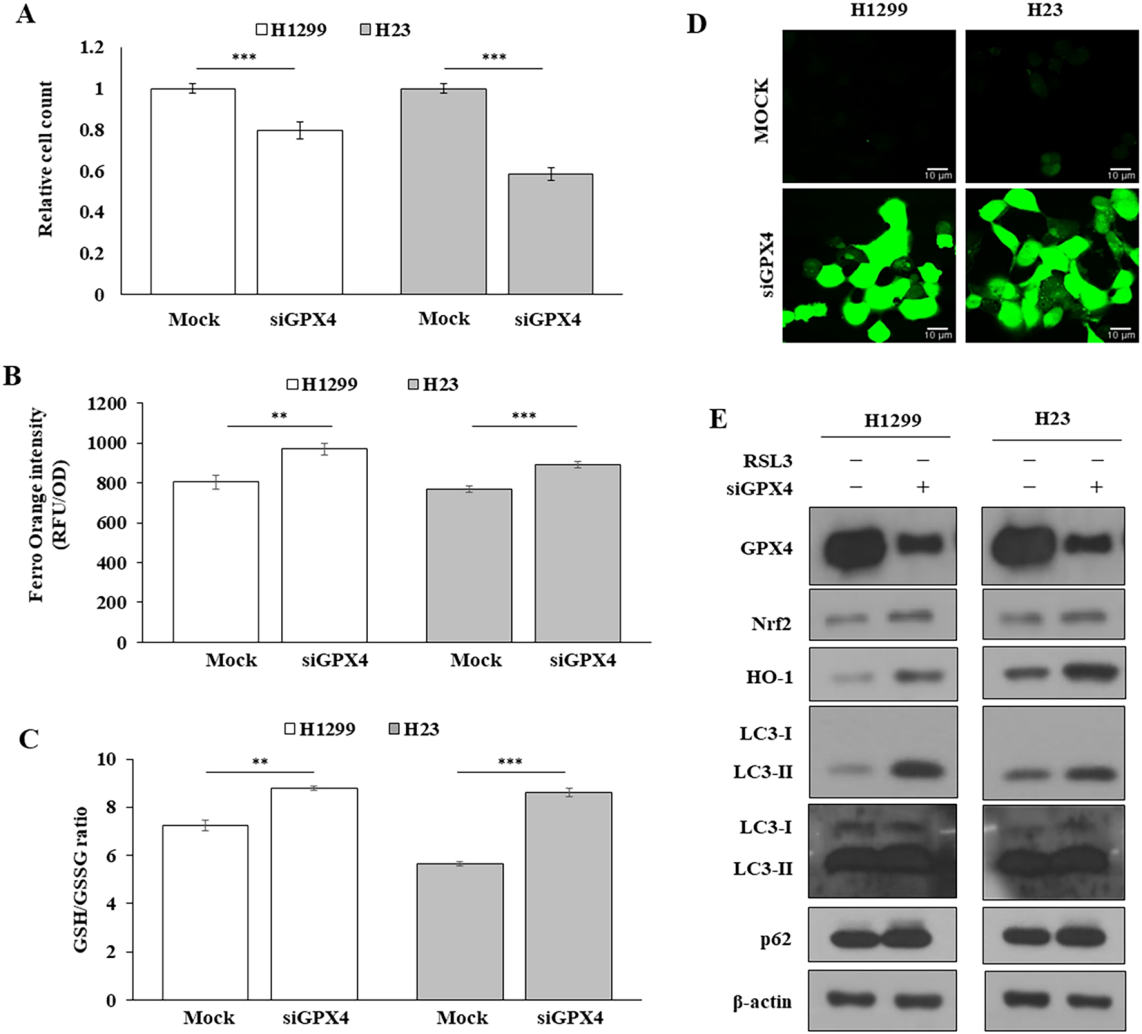
Knockdown of GPX4 (siGPX4) had an effect similar to RSL3 treatment. (**A**) Cell proliferation examined by cell counting. (**B**) LIP level measured by the fluorescence intensity of FerroOrange. (**C**) GSH/GSSH ratio was determined by an EZ-Glutathione assay kit. (**D**) Cellular ROS determined using DCFA/H2DCFDA-cellular ROS assay kit by confocal microscope (scale bar: 10 µm). (**E**) GPX4 expression detected by western blotting. *p < 0.05, **p < 0.01, ***p < 0.001 compared with control.

Since RSL3 suppressed GPX4, the effects of GPX4 knockdown on cell proliferation, LIP levels, the GSH/GSSH ratio, and ROS levels in H1299 and H23 cells were examined. GPX4 knockdown reduced cell proliferation and significantly increased LIP levels, the GSH/GSSG ratio, and ROS levels when compared to the control group (Fig. 5A–D). The Nrf2/HO1 ratio increased, consistent with the RSL3 treatment (Fig. 5E). These results suggested that GPX4 is a key molecule associated with the Nrf2/HO1 pathway for ferroptosis in RSL3-sensitive NSCLC.

### RSL3 inhibited NSCLC tumor growth in a zebrafish model

**Figure 6:**
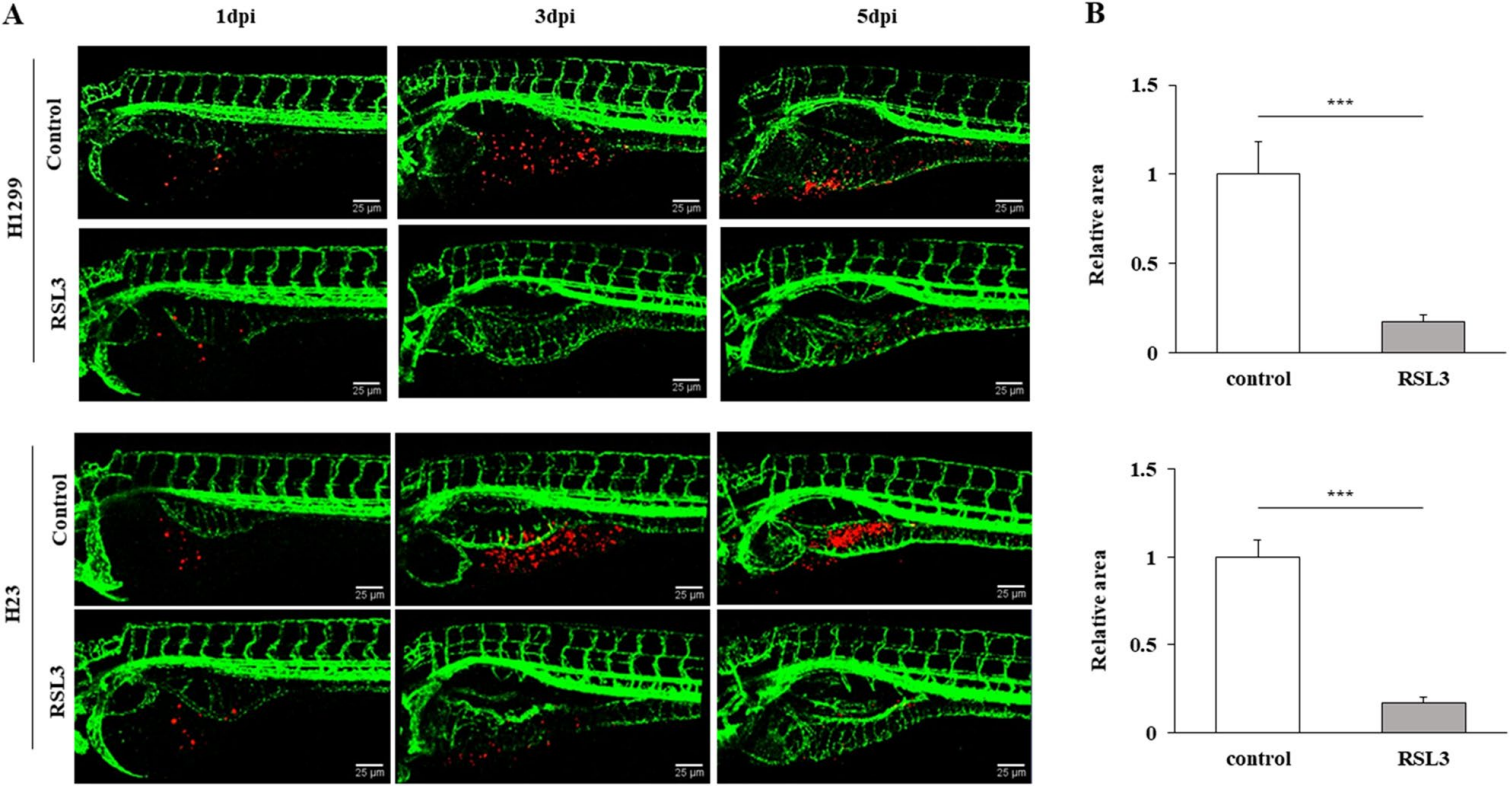
RSL3 treatment had an anticancer effect that was specific to cancer cells in vivo. (**A**) Representative confocal images of CM-Dil-labeled cancer cells (red) in the vasculature (green) of zebrafish larvae (scale bar: 25 µm). (**B**) Relative area penetrated by CM-Dil-labeled cancer cells after treatment with control or RSL3. Results are shown as the mean ± SD of triplicate experiments. *p < 0.05, **p < 0.01, ***p < 0.001 compared with control.

Suppression of tumorigenicity by RSL3 treatment was examined in xenograft zebrafish models using CM-Dil-labeled H1299 or H23 cells. The cells (red) were grafted onto *Tg(flk1:EGFP)* zebrafish embryos and treated with E3 medium containing either DMSO or RSL3 for 5 days. RSL3 treatment reduced the tumor area in both the H1299 and H23 cell xenografts (Fig. 6A,B). This indicates that RSL3 has anticancer effects, which is consistent with the in vitro results.

## Discussion

Lung cancer is a leading cause of cancer-related deaths worldwide. The most common type of lung cancer is NSCLC, accounting for approximately 80% of all lung cancers. Treatment of NSCLC involves a multidisciplinary approach, which consists of surgery, radiotherapy, and systemic treatment with various types of drugs, including chemotherapeutics, targeted therapies, and immune checkpoint inhibitors. Recent developments in targeted therapy and immunotherapy have changed treatment process for NSCLC^27^. However, the mutation required for targeted therapy did not exist in any patient. In addition, because drug resistance can occur to various agents, there is a need to identify and develop novel agents for the treatment of NSCLC.

Cancer therapeutics have long been associated with cell death because many chemotherapeutic agents have been designed to promote the death of malignant cells. Ferroptosis is a unique, non-apoptotic type of cell death that has recently been discovered; thus, ferroptosis-inducing agents have been investigated for use in NSCLC treatment^29,30^. Recently, the poor prognostic value of upregulation of ferroptosis associated genes SLC7A11 and GPX4 has also been reported in lung cancer^31,32^. However, studies have examined the effects of erastin and RSL3 on NSCLC cells are very limited. Erastin has been shown to inhibit SLC7A11, which functions in the Nrf2/Keap1 pathway in N5CP and A549 cells^33,34^, and sensitizes NSCLC cells to the chemotherapeutic drug celastrol^35^. RSL3 inhibits GPX4 activity in H1650, HCC827, and PC9 cells^36^. ALOX15, a member of the lipoxygenase family, downregulates Calu-1 cells and increases sensitivity to both erastin and RSL3^14^. Moreover, these cytotoxic effects on NSCLC cells have not been evaluated in normal cells, which is very important. In this study, we evaluated whether erastin and RSL3 induce cell death in NSCLC cells compared to that in normal bronchial epithelial cells, BEAS-2B. Although both erastin and RSL3-induced cell death in all the NSCLC cells tested, only RSL3 showed minimal cytotoxic effects on BEAS-2B cells. Comparison of IC_50_ showed that RSL3 was a more effective compound for inducing cell death than erastin (Table 1). In addition, RSL3 showed varying levels of sensitivity in different NSCLC cells, which showed a significant association with the endogenous expression of GPX4, a target of RSL3. We verified that RSL3 induces ferroptosis via GPX4 inhibition and Nrf2/HO1 induction. HO1, a target of Nrf2, is a cytoprotective enzyme that degrades hemoglobin into CO, iron, and biliverdin^37^, and its function in ferroptosis is contradictory, suggesting that dual, protective, or detrimental effects depend on the degree of cellular iron levels and ROS, indicating enhanced ferroptosis via the Nrf2/HO1 axis in our study^23,38^. This effect was recovered only by ferrostatin-1, a ferroptosis inhibitor, and not by the other programmed cell death inhibitors examined. Further studies confirmed that GPX4 knockdown induces Nrf2/HO1 expression, increases lipid peroxidation and cellular LIP levels, and inhibits GSH-to-GSSH conversion. These results indicate that the inhibition of GPX4 expression along with Nrf2/HO1 activation is the main factor in RSL3-induced ferroptosis in NSCLC cells, depending on the endogenous levels of GPX4. This may be because GPX4 is necessary or has a more functional role in the GPX4 overexpressing cancer cell, making the cells sensitive to RSL3. Furthermore, an in vivo zebrafish xenograft model confirmed the suppression of tumor growth by RSL3 in NSCLC cells.

Emerging studies have revealed the crosstalk between autophagy and ferroptosis. However, the role of autophagy in ferroptosis is controversial, as autophagy activation can either induce or inhibit ferroptosis, although several studies have shown that ferroptosis induction is associated with autophagy activation^39–41^. Our examination of autophagic flux showed an increase in autophagosomes, but not autolysosomes, along with the accumulation of both LC3-II and p62 by RSL3, indicating the inhibition of autophagic flux. Although it was not able to clearly elucidate the mechanism underlying the decrease in autolysosomes relative to autophagosomes in this moment, it can be hypothesized that the fusion of autophagosomes with lysosomes may be disrupted due to lysosome dysregulation. Thus, lysosomal dysregulation was verified by the release of cathepsin B into the cytoplasm with enlarged lysosomes and massively increased acidic compartments, indicating impaired lysosomal membrane stability, which points to the lysomotropic effect of RSL3^42^. These results are consistent with recent reports suggesting an association between lysosomal dysregulation and ferroptosis^43–45^. However, the detailed mechanism needs to be further investigated.

Overall, the results of this study suggest that RSL3 is a more effective ferroptotic cell death inducer than erastin in NSCLC cells, and it has very limited cytotoxic effects on normal cells. Because the activity of RSL3 is dependent on GPX4 expression levels, predictions can be made regarding whether ferroptosis will occur in NSCLC cells. Further research is required to investigate the synergistic effect of RSL3 with other targeted or immunotherapy agents, as well as the effect of RSL3 on EGFR-TKI-resistant NSCLC cells.

## Materials and methods

### Cell culture and reagents

Human NSCLC (HCC827, H1299, H23, H820, H1975, PC9, H1573, H1793, H460, and A549) and normal human bronchial epithelial (BEAS-2B) cell lines were purchased from the American Type Culture Collection (Manassas, VA, USA). All cells were grown and maintained in RPMI-1640 medium supplemented with 10% fetal bovine serum (FBS) and 1% penicillin/streptomycin (Welgene, Gyeongsangbuk-do, South Korea) at 37 °C in a humidified atmosphere containing 5% CO_2_. RSL3, ferrostatin-1, Z-VAD-FMK, necrostatin-1 and etoposide were purchased from Selleck Chemicals (TX, USA). Chloroquine diphosphate salt, bafilomycin A1, and acridine orange were purchased from Sigma-Aldrich (St. Louis, MO, USA). The compounds were dissolved in dimethyl sulfoxide (DMSO, Sigma-Aldrich). The oligonucleotide sequences of human *GPX4* siRNA (siGPX4) were as follows: 5′-UUCGAUAUGUUCAGCAAGAUU-3′ (sense) and 5′-UCUUGCUGAACAUAUCGAAUU-3′ (antisense) (Shanghai GenePharma Co. Ltd., Shanghai, China).

### Cell viability and proliferation assays

Cell viability was assessed using the 3-(4,5-dimethylthiazol-2-yl)-2,5-diphenyltetrazolium bromide (MTT) assay (Sigma-Aldrich). Cells were seeded in 96 well culture plates at a density of 1 × 10^3^ cells/well and treated with the indicated concentrations of erastin, RSL3, ferrostatin-1, Z-VAD-FMK, chloroquine diphosphate salt, bafilomycin A1, and necrostatin-1. After incubation, 10 µL MTT solution (0.5 mg/mL) dissolved in distilled water and 20 µL medium was added to each well. After 2 h of additional incubation, the MTT solution was discarded and DMSO was added. Absorbance was measured at 570 nm using a microplate reader (SpectraMax Plus 384, CA, USA). Cells were counted to measure cell proliferation. Cells were seeded in 60 mm dishes at a density of 2 × 10^5^ cells/dish. The following day, cells were treated with either 100 nM RSL3 or 1 μM ferrostatin-1 for 24 h. The cells were counted using a hemocytometer after trypan blue staining (Sigma-Aldrich). Cells were imaged using an inverted light microscope (Olympus IX71, Olympus Corp., Japan).

### RNA isolation and quantitative real-time PCR (qRT-PCR)

Total RNA was extracted from the cells using TRIzol reagent (Invitrogen, CA, USA). cDNA was synthesized from total RNA using a reverse transcription kit (Lapopass; Cosmo Genetech, Seoul, South Korea) following the manufacturer’s instructions. qRT-PCR was performed using SYBR Green Q Master Mix (Labopass) and a QuantStudio 3 Real-Time PCR Instrument (Applied Biosystems, MA, USA). The oligonucleotide primers that were used for qRT-PCR are as follows; *GPX4* sense:5′-AGAGATCAAAGAGTTCGCCGC-3′, antisense:5′-TCTTCATCCACTTCCACA GCG-3′; *GAPDH* sense:5′-ACCCACTCCTCCACCTTTGA-3′, antisense:5′-CTGTTGCTG TAGCCAAATTCGT-3′. Ct values for *GPX4* were normalized to that of *GAPDH*. Each variable was assessed in triplicates and repeated in three independent experiments.

### Immunoblotting

Cells were lysed in RIPA buffer that contained a protease/phosphatase inhibitor cocktail (Sigma-Aldrich) at 4 °C for 30 min and used for immunoblotting. The cytosolic fraction for cathepsin B detection was prepared as previously described^45^. A total of 20 μg of protein was loaded per lane of an SDS-PAGE gel and separated by electrophoresis. Proteins were transferred onto nitrocellulose (GE Healthcare, IL, USA) or PVDF membranes (IPVH00010, Millipore, MA, USA). Membranes were subsequently probed with primary antibodies and incubated with either goat anti-mouse IgG (Cell Signalling, 7076, MA, USA) or goat anti-rabbit IgG (Cell Signaling,7074) secondary antibodies conjugated with horseradish peroxidase (HRP). Chemiluminescence was detected using an enhanced chemiluminescence (ECL) system (Translab). The following primary antibodies were used: GPX4 (ab125066, Abcam), SCL7A11 (Cell Signalling, 12,691), Nrf2 (ab62352, Abcam), Keap1 (Cell Signalling, 8047), HO-1 (Cell Signalling, 5853), p62 (ab56416, Abcam), LC3 (PM036, MBL), cathepsin B (Cell Signalling, 31,718) and β-actin (Santa Cruz Biotechnology, sc-47778). The result of gels images was cropped and full-length gels and blots are included in the Supplementary Data.

### Flow cytometry

Cell death was measured using the FITC Annexin V Apoptosis Detection Kit I (BD Pharmingen, NJ, USA), following the manufacturer’s instructions. The cells were seeded at a density of 2 × 10^5^ in 60 mm dishes. The following day, cells were treated with RSL3 (100 nM) and/or ferrostatin-1 (1 µM) for 24 h and then the cells were harvested with culture medium and washed twice with cold PBS. The pellet was resuspended in 200 μL of ice-cold binding buffer and labeled with 5 µL FITC annexin V and 5 µL PI for 15 min at 20 °C in the dark. Cell death was analyzed using a flow cytometer (BD Biosciences, BD FACSCanto™ II Flow Cytometry System, NJ, USA).

### Labile ferrous ion pool (LIP) analysis

The intracellular labile iron (II) pool (LIP) was measured using a FerroOrange kit (F374; Dojindo, Kumamoto, Japan) following the manufacturer’s instructions. FerroOrange is a fluorescent probe that detects labile iron (II) ions (Fe^2+^) only. The cells were seeded in 96 well plates at a density of 1 × 10^4^ cells/well and treated with RSL3 or ferrostatin-1 for 24 h. The culture medium was then removed, and the cells were washed with serum-free RPMI-1640 medium. The cells were incubated with 1 µM FerroOrange in serum-free RPMI-1640 at 37 °C for 30 min. FerroOrange intensity was measured at excitation and emission wavelengths of 543 and 580 nm, respectively, using an EnSpire Multi Plate Reader (PerkinElmer, MA, USA). Each experiment was performed in triplicate and repeated three times.

### Lipid peroxidation and ROS analysis

Lipid peroxidation activity in the cells was analyzed using BODIPY 581/591 C11 (Invitrogen, D3861, MA, USA). For live cell imaging, cells were seeded in a confocal dish at a density of 1 × 10^4^. The following day, the cells were treated with either RSL3 (100 nM) or ferrostatin-1 (1 μM) for 24 h. Next, the cells were incubated in fresh medium with BODIPY 581/591 C11 (2 μM) and covered with a sterile coverslip before incubation at 37 °C for 30 min. BODIPY fluorescence was measured at 20 °C using the LSM 780 NLO system (Carl Zeiss, Baden-Württemberg, Germany). Fluorescence intensity was analyzed using ZEN software (Carl Zeiss, Baden-Württemberg, Germany). Cellular ROS levels were analyzed using a DCFA/H2DCFDA-cellular ROS assay kit (ab11385, Abcam) after treatment with RSL3 for 6 h following the manufacturer’s instructions. Absorbance was measured at excitation and emission wavelengths of 543 and 580 nm, respectively, using an Enspire Multi Plate Reader (PerkinElmer, MA, USA). ROS generation in live cells treated with DCFDA was measured using an LSM 780 NLO (Carl Zeiss, Baden-Württemberg, Germany). 

### GSH/GSSG analysis

The GSH concentration and GSH/GSSG ratio were calculated using the EZ-Glutathione Assay Kit (Dogenbio, Seoul, South Korea) following the manufacturer’s instructions. Cells were seeded at a density of 5 × 10^5^ cells/dish in 60 mm dishes. The following day, the cells were treated with RSL3 and ferrostatin-1 for 24 h. Cells were harvested and sonicated with PBS, and cell debris was removed. Absorbance was measured at 412 nm using a microplate reader (SpectraMax Plus 384). Each experiment was performed in triplicate and repeated three times.

### Autophagic flux assessment

Autophagic flux was assessed in live cells by mRFP-GFP-LC3 expressing plasmid, which was kindly provided by Professor T. Yoshimori of the Department of Genetics, Graduate School of Medicine, Osaka University, Japan^46^. Cells cultured on confocal dishes were transfected with mRFP-GFP-LC3 plasmid for 24 h and then treated with or without RSL3 for 24 h. Fluorescence images were obtained using a Laser Scanning Confocal Microscope (LSM800, Carl Zeiss) and visualized with a 63 × water immersion objective lens.

### Immunofluorescence staining

The cells were fixed and stained with primary antibodies against LAMP-1 (sc-20011, Santa Cruz Biotechnology) and cathepsin B, followed by a secondary antibody conjugated with Alexa Fluor 488 or 546. Nuclei were counterstained with (DAPI; 10236276001; Roche, Mannheim, Germany)**.** Live cells were stained with 5 μg/mL acridine orange at 37 ℃ for 30 min. Fluorescent images were obtained using a laser-scanning confocal microscope (LSM800; Carl Zeiss, Oberkochen, Germany).

### In vivo* zebrafish tumor model*

Zebrafish (*Danio rerio*) and embryos were bred and maintained according to standard procedures. All animal experimental protocols were approved by the Committee for Ethics of Animal Experimentation of Sookmyung Women’s University (SMWU-IACUC-1712–036-03) and were performed in accordance with the relevant guidelines and regulation of Sookmyung Women's University as previously described^47^. The authors confirm that all animal experiments in the present study have been performed in accordance with ARRIVE guidelines. Approximately 50 fluorescent cell tracker CM-Dil-labeled H1299 or H23 cells were injected into the yolk sac of zebrafish embryos at 2 days post-fertilization (dpf). One day post injection (dpi), the zebrafish larvae were treated with RSL3 (100 nM) and maintained at 34 °C for 5 days. Fluorescence images were acquired using a Zeiss LSM700 confocal microscope (Carl Zeiss AG). The area penetrated by the CM‑Dil-labeled cancer cells was quantified using the ImageJ software (version 1.52n, NIH) and normalized to cancer cells in untreated zebrafish embryos for each group.

### Statistical analysis

SPSS Statistics version 25 software (IBM, NY, USA) and GraphPad Prism version 5 software (GraphPad, CA, USA) were used for statistical analyses. Experimental results were presented as the mean ± SD from at least two independent experiments. *T*-tests were performed to compare two independent groups. Comparisons of nultiple groups were analyzed using ANOVA. Significance was defined as: # = 0.05 < p (No significance); * = 0.01 < *p* < 0.05; ** = 0.001 < *p* < 0.01; *** = *p* < 0.001.

## Supplementary Information

Supplementary Information.

## Data Availability

The raw scanned bot images used in the figures are provided in Supplementary Data. Other datasets used and/or analyzed in the current study are available from the corresponding author upon request.
