# Supplementary material for: GPX4 overexpressed non-small cell lung cancer cells are sensitive to RSL3-induced ferroptosis
Source: Sci Rep. 2023 May 31;13:8872. doi: 10.1038/s41598-023-35978-9 (PMC10232506; doi:10.1038/s41598-023-35978-9)
Supplement: Supplementary file 1 — Supplementary Information. [file 41598_2023_35978_MOESM1_ESM.pdf]

Supplementary Data

GPX4 overexpressed non-small cell lung cancer cells are sensitive to RSL3-induced ferroptosis

Joo-Won Kim1,2+, Dong Wha Min1,2+, Dasom Kim1,2, Joohee Kim3, Min Jung Kim3, Hyangsoon Lim1, Ji-Yun Lee1*

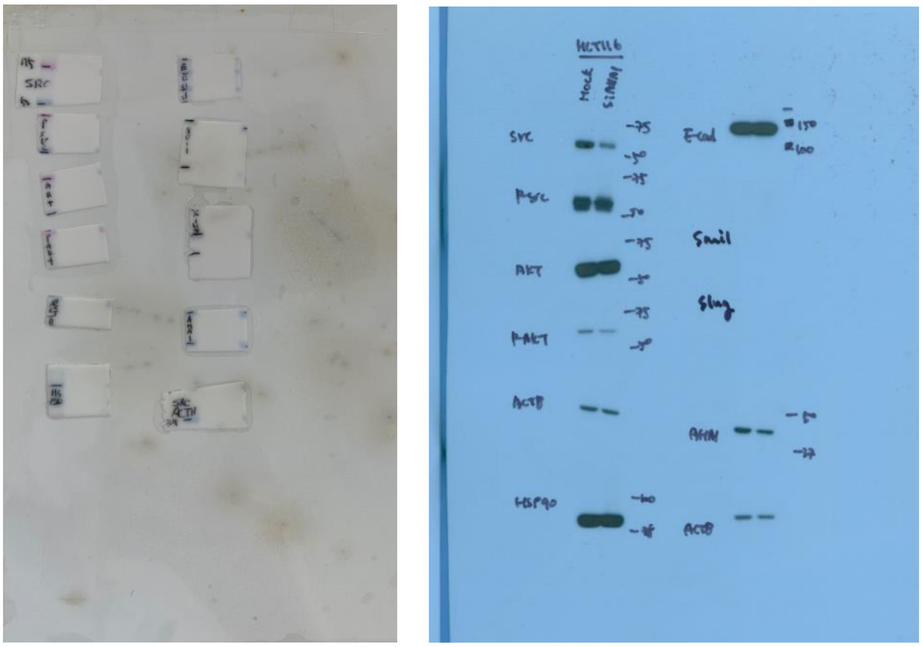

Fig. S1. Example of membrane cut of target protein to probe with the appropriate primary antibody. Image in the supplementary data is from original, unprocessed blot. We cut the blot prior to hybridization with antibodies, and detect different blot from other projects in the same film, which is not published. Because of these reasons, we covered up some area and cropped film. Data from at least three different blot films were shown in below supplementary figures.

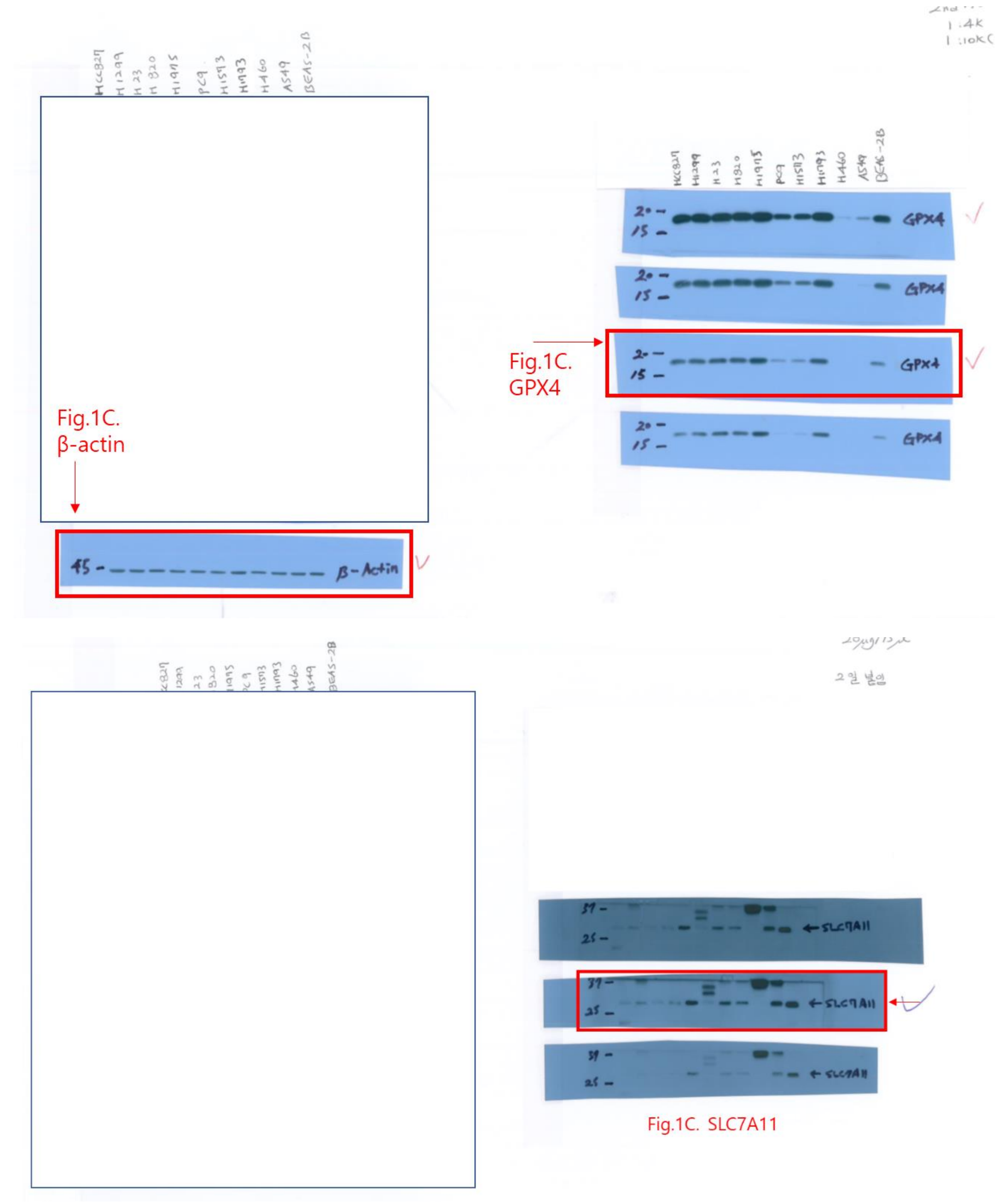

Fig. S2. Raw images of fig. 1C

A

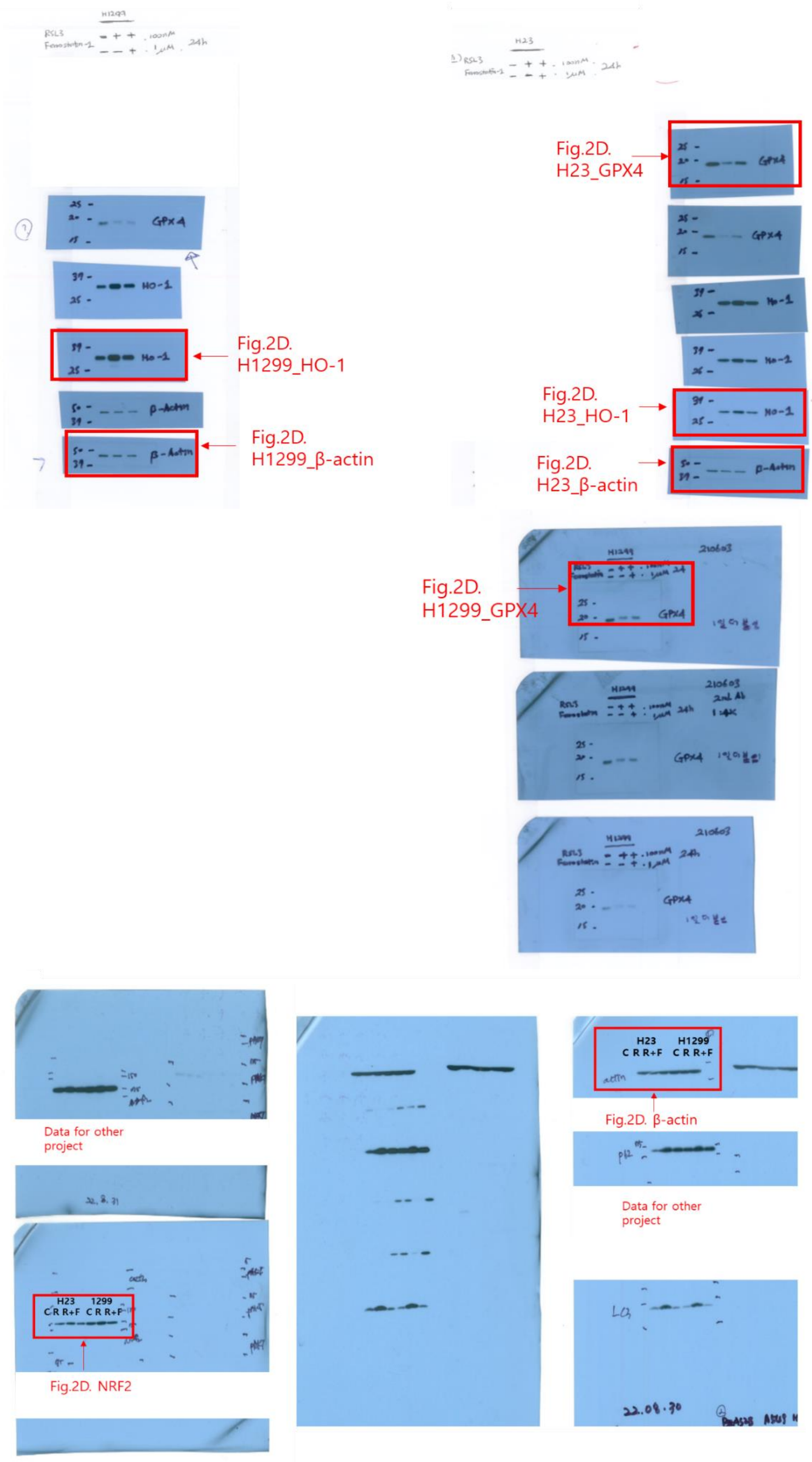
Fig.2D. H23_GPX4
Fig.2D. H1299_HO-1
Fig.2D. H1299_β-actin
Fig.2D. H23_HO-1
Fig.2D. H23_β-actin
Fig.2D. H1299_GPX4
Data for other project
H23 1299
C R R+F C R R+F
Fig.2D. NRF2
H23 H1299
C R R+F C R R+F
Fig.2D. β-actin
Data for other project

B

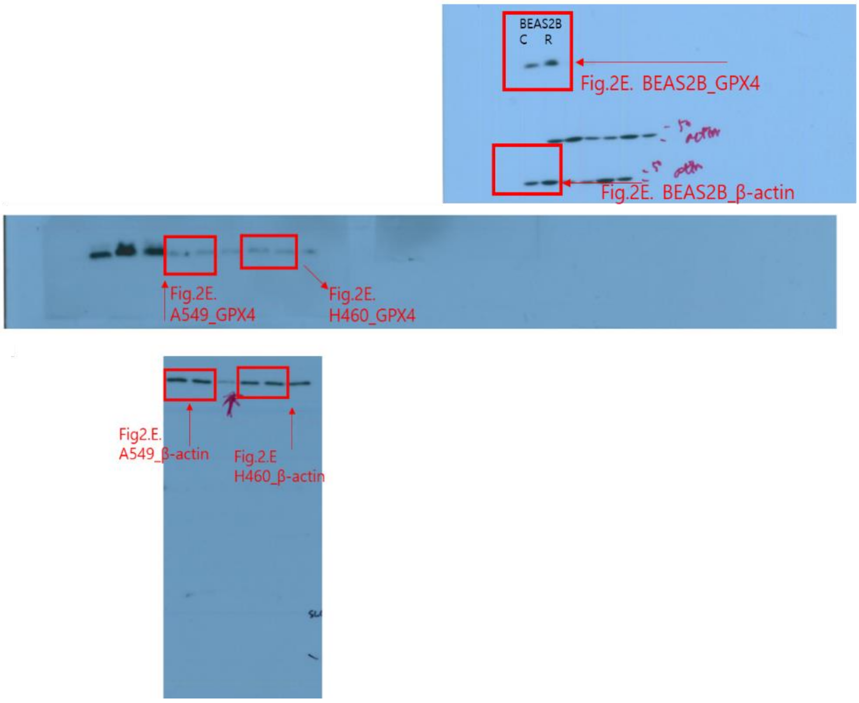
BEAS2B
C R
Fig.2E. BEAS2B_GPX4
Fig.2E. BEAS2B_β-actin
Fig.2E.
A549_GPX4
Fig.2E.
H460_GPX4
Fig2.E.
A549_β-actin
Fig.2.E
H460_β-actin

C

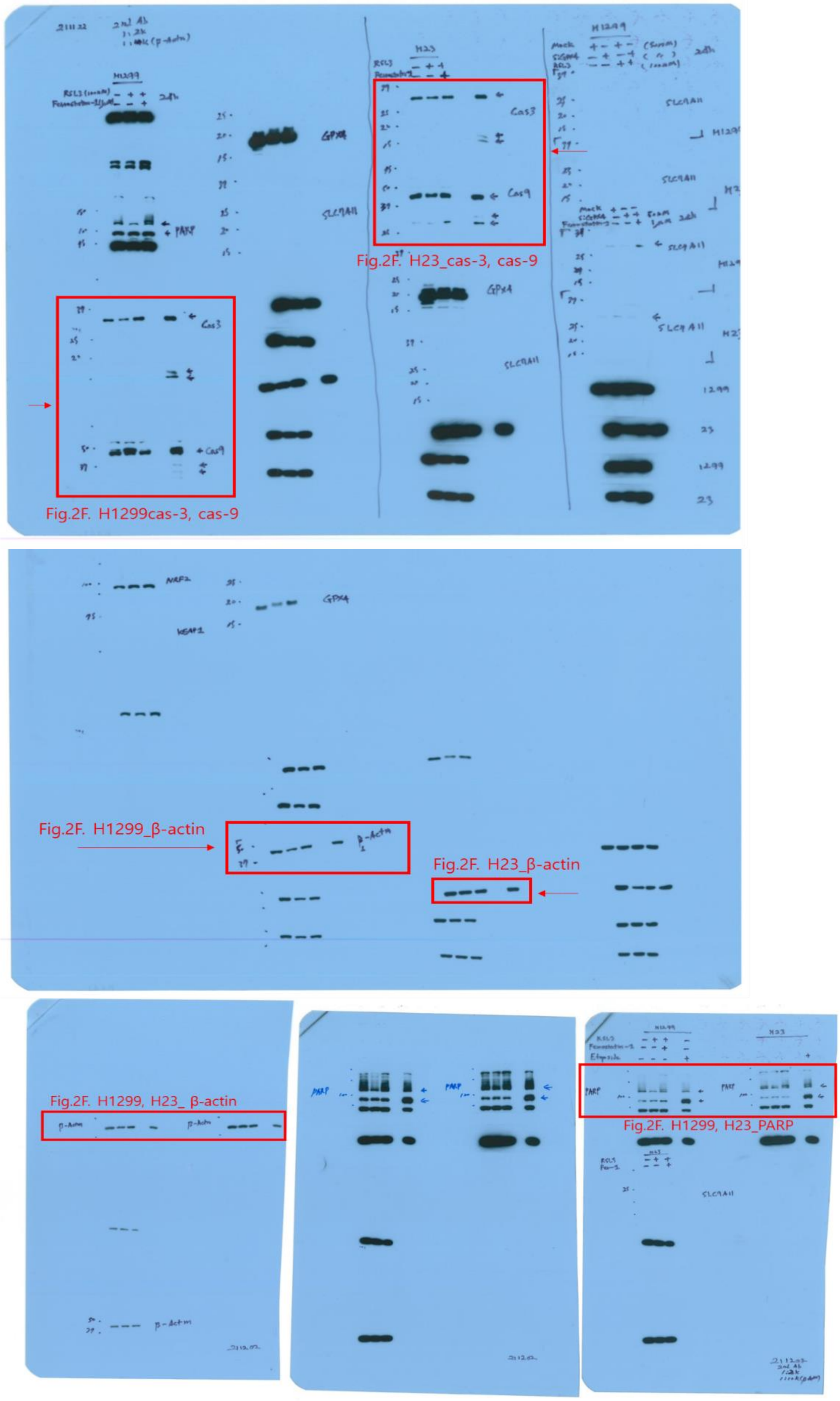

Fig. S3. Raw images of fig. 2D (A), E (B), and F (C)

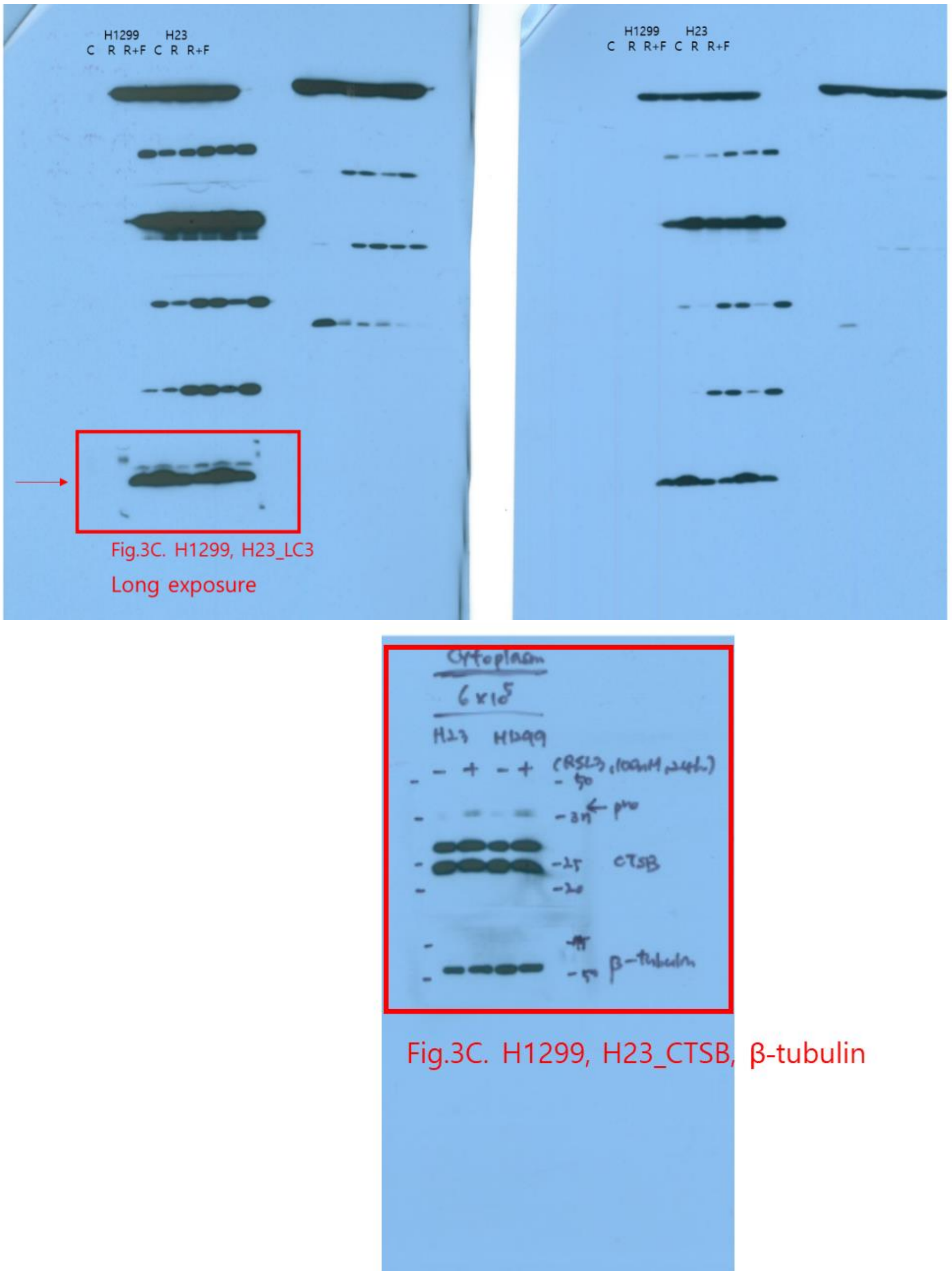

Fig. S4. Raw image of fig. 3C

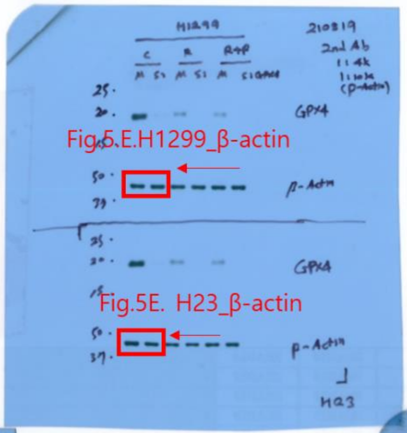
H1299
210819
2nd Ab
GPX4
Fig.5.E.H1299_β-actin
β-Actin
GPX4
Fig.5E. H23_β-actin
β-Actin
H23

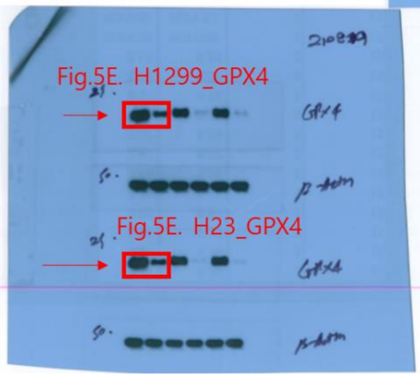
210819
Fig.5E. H1299_GPX4
GPX4
β-Actin
Fig.5E. H23_GPX4
GPX4
β-Actin

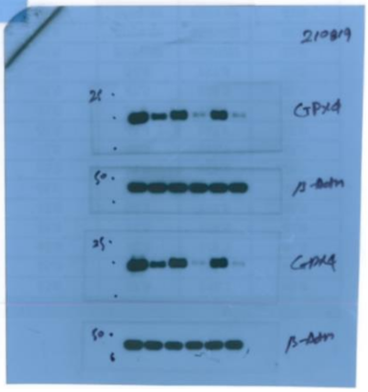
210819
GPX4
β-Actin
GPX4
β-Actin

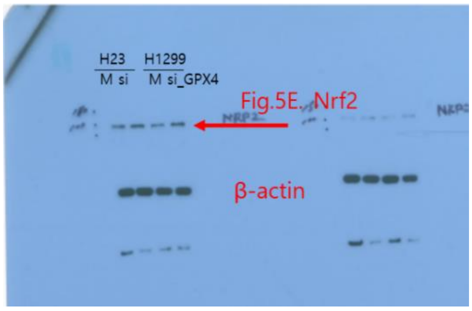
H23
M si
H1299
M si_GPX4
Fig.5E. Nrf2
β-actin

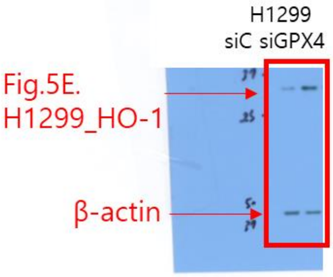
H1299
siC siGPX4
Fig.5E.
H1299_HO-1
β-actin

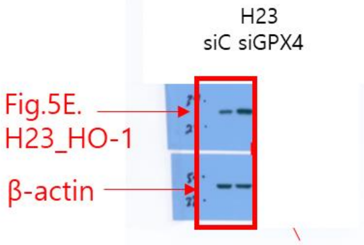
H23
siC siGPX4
Fig.5E.
H23_HO-1
β-actin

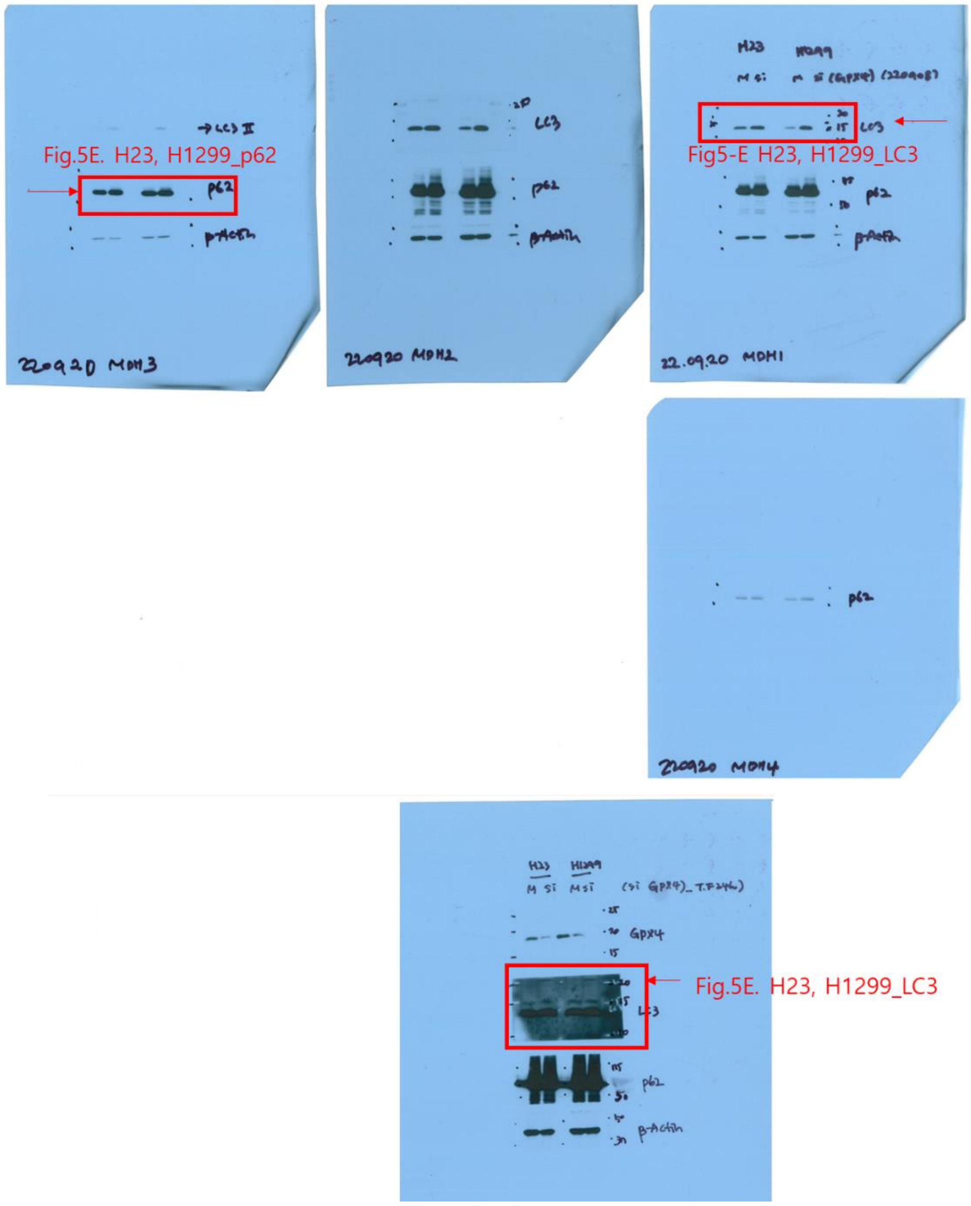

Fig. S5. Raw images of fig. 5E

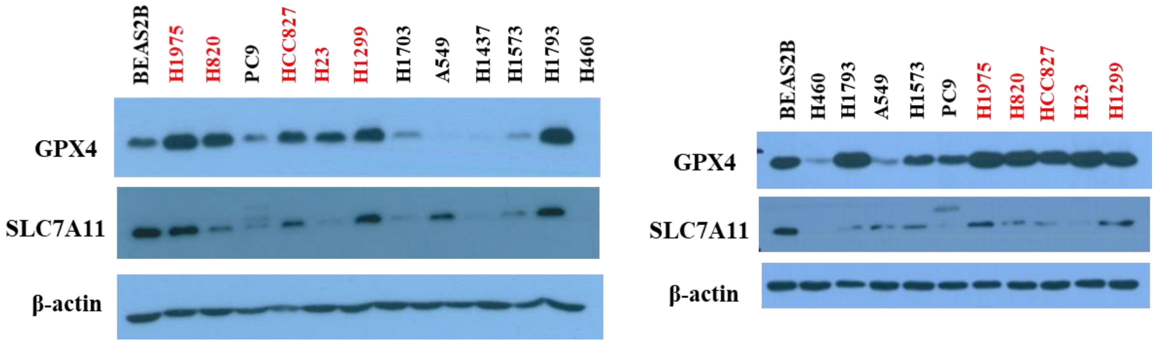

Fig. S6. Additional raw images support fig. 1C

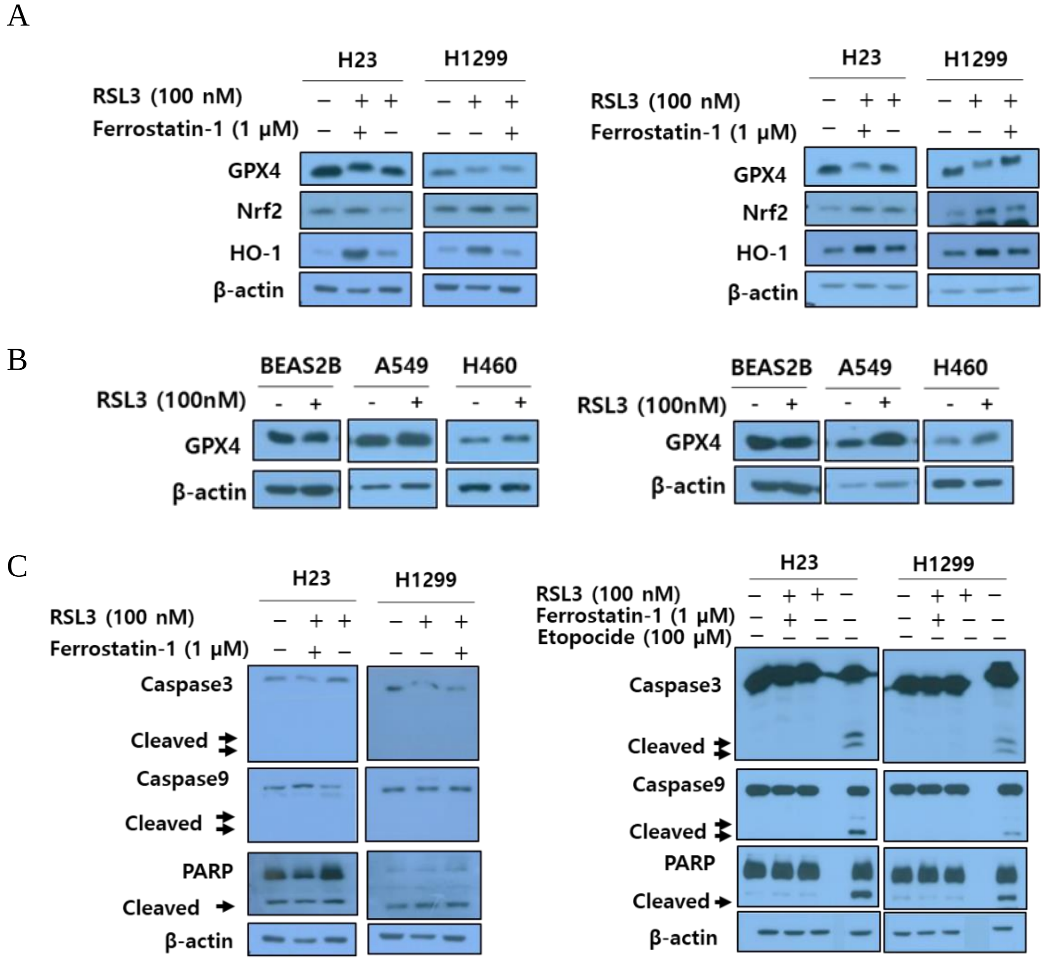

Fig. S7. Additional raw image support fig. 2D (A), E (B), F (C)

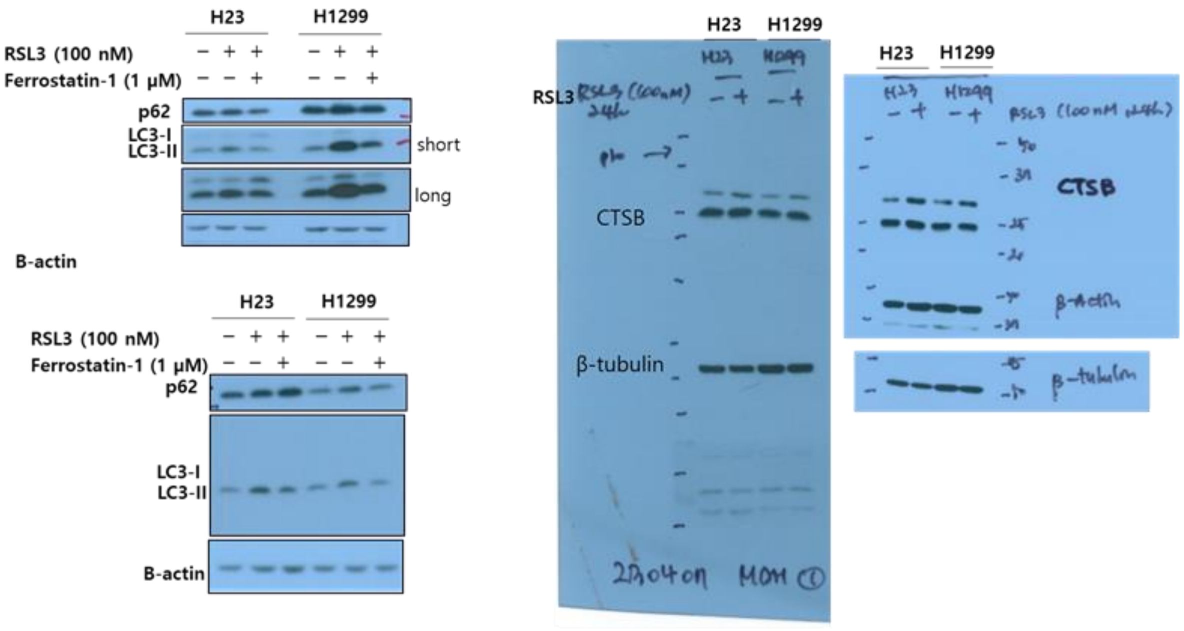

Fig. S8. Additional raw images support fig. 3C

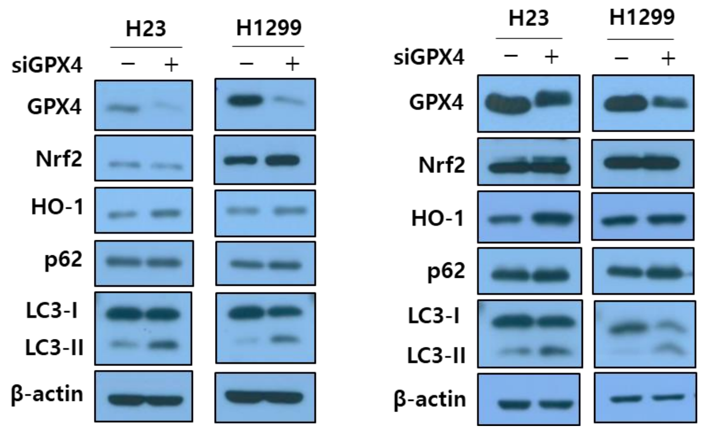

Fig. S9. Additional raw images support fig. 5E
